# Evolution and exchange of plasmids in pathogenic *Neisseria*

**DOI:** 10.1128/msphere.00441-23

**Published:** 2023-10-18

**Authors:** Wearn-Xin Yee, Tabea Elsener, Ana Cehovin, Martin C. J. Maiden, Christoph M. Tang

**Affiliations:** 1 Sir William Dunn School of Pathology, University of Oxford, Oxford, United Kingdom; 2 Department of Biology, University of Oxford, Oxford, United Kingdom; Antimicrobial Development Specialists, LLC, Nyack, New York, USA

**Keywords:** evolution, plasmid, *Neisseria*, conjugation, AMR, HGT

## Abstract

*Neisseria gonorrhoeae* causes sexually transmitted infection (STI) gonorrhoea and is on the WHO critical list due to increasing antimicrobial resistance (AMR). The bacterium can carry a conjugative plasmid, pConj, which prevents the use of tetracycline or doxycycline for treating gonorrhea, and is responsible for spread of a β-lactamase plasmid, p*bla*; over 90% of gonococcal isolates also harbor a small cryptic plasmid, pCryp. We systematically analyzed the presence of these plasmids in other *Neisseria* spp., including *Neisseria meningitidis*, which causes sepsis/meningitis. pConj is the most frequently found plasmid in the meningococcus and is in many clonal complexes. The plasmid is associated with meningococcal carriage rather than disease, indicating that pConj imposes fitness costs during systemic disease. Phylogenetic analyses reveal that pConj is genetically diverse in *N. meningitidis*, indicating that it shares a long evolutionary history with the meningococcus and that the plasmid has been transferred at least twice from *N. meningitidis* to *N. gonorrhoeae*. Following the first transfer, gonococcal isolates carrying the plasmid underwent clonal expansion and disseminated pConj to other gonococcal lineages. The second introduction was associated with an altered conjugation machinery which reduces conjugation efficiency. Therefore, in contrast to chromosomal resistance which has evolved through introduction of genes from commensals, gonococcal plasmid-mediated resistance has arisen through transfer from another pathogen, *N. meningitidis*. Further antibiotic pressure from the use of doxycycline for post-exposure prophylaxis against STIs is likely to promote plasmid-mediated AMR in both *N. gonorrhoeae* and *N. meningitidis*.

**IMPORTANCE:**

Horizontal gene transfer (HGT) is a major influence in driving the spread of antimicrobial resistance (AMR) in many bacteria. A conjugative plasmid which is widespread in *Neisseria gonorrhoeae*, pConj, prevented the use of tetracycline/doxycycline for treating gonococcal infection. Here, we show that pConj evolved in the related pathogen, *Neisseria meningitidis*, and has been acquired by the gonococcus from the meningococcus on multiple occasions. Following its initial acquisition, pConj spread to different gonococcal lineages; changes in the plasmid’s conjugation machinery associated with another transfer event limit spread in the gonococcal populations. Our findings have important implications for the use of doxycycline to prevent bacterial sexually transmitted disease which is likely to exacerbate the spread of AMR through HGT in pathogenic bacteria.

## INTRODUCTION

*Neisseria gonorrhoeae* causes the sexually transmitted infection (STI) gonorrhoea, which is a serious public health concern due to the emergence of antimicrobial resistance (AMR). Horizontal gene transfer (HGT) is a major contributing factor in the development of AMR in *N. gonorrhoeae*. It is becoming increasingly appreciated that commensal *Neisseria* spp. are the main source of chromosomal resistance determinants in the gonococcus (1). For example, the acquisition of *penA* alleles by transformation leads to penicillin and cephalosporin resistance in the gonococcus (2), while mosaic alleles encoding the MtrCDE export pump contribute to azithromycin resistance (3).

In addition to chromosomally mediated AMR, *N. gonorrhoeae* can be resistant to penicillin and tetracycline through a 3.2- to 9.3-kb β-lactamase plasmid, p*bla*, and a 39- to 43-kb conjugative plasmid, pConj, respectively (4, 5). Importantly, pConj is found in around a third of gonococci (6) and can contain *tetM*, which likely originated from a streptococcal transposon (5) and is responsible for high-level resistance against doxycycline as well as tetracycline (6). Therefore, the spread of pConj is likely to undermine the use of doxycycline in post-exposure prophylaxis (doxy-PEP) which is being evaluated for preventing bacterial STIs following unprotected sex (7).

Variants of p*bla* and pConj are present in the gonococcal population (6). Specifically, seven variants of pConj which differ in the presence of *tetM* and other genes including those encoding toxin:antitoxin (TA) systems (6) have been described, while three versions of p*bla* occur commonly (8). pConj encodes a Type IV secretion system (T4SS) (9) that enables conjugation and spread within gonococcal populations, as well as between the gonococcus and other species (10). Furthermore, pConj mediates the mobilization of p*bla* (11). Over 90% of gonococcal isolates also harbor pCryp, a 4.2-kb plasmid of unknown function (6, 12).

pConj and p*bla* are also found in other *Neisseria* spp., including *Neisseria meningitidis*, a leading cause of bacterial meningitis and sepsis (13). *N. gonorrhoeae* and *N. meningitidis* are human-specific pathogens that usually occupy distinct anatomical sites, the urogenital and oropharyngeal tracts, respectively (14). However, the gonococcus occasionally colonizes the nasopharynx where it can acquire AMR genes from commensal *Neisseria* (2, 3), while some strains of *N. meningitidis* asymptomatically colonize the urethra and cause a syndrome indistinguishable from gonococcal disease (15). This provides opportunities for the exchange of plasmids between the two pathogens; indeed, in the laboratory, pConj and p*bla* can be transferred by conjugation from *N. gonorrhoeae* into *N. meningitidis* and subsequently between meningococcal strains (10, 11, 16, 17). Additionally, case reports have described *N. meningitidis* containing p*bla* and/or pConj (18), including those causing invasive meningococcal disease (IMD) (19), while pCryp has also been found in *N. meningitidis* (20). Finally, *N. meningitidis* isolates from urogenital tract and throat have been found with *tetM^+^* pConj but not p*bla* (17, 21–23).

Due to the public health threat posed by AMR plasmids (24), we systematically examined the presence, distribution, and evolutionary relationships of the gonococcal plasmids, pConj, p*bla*, and pCryp in *N. meningitidis* and commensal *Neisseria* spp. We interrogated over 19,000 publicly available meningococcal whole genome sequences (WGS) on PubMLST (25) for the presence of these plasmids. Although a limited number of *N. meningitidis* strains contain p*bla* or pCryp, we found that ~1 in 200 meningococcal isolates carry pConj. There are only a few instances of this plasmid in commensals given the relative lack of WGS data. pConj in *N. meningitidis* is much more genetically diverse than the plasmid in the gonococcus; much of the variation occurs as insertions and deletions in the 9- to 13-kb genetic load (GL) region which harbors genes involved in plasmid maintenance +/-*tetM* (9, 26); however, even after we eliminated the GL region from analysis (as large-scale recombination distorts phylogenetic relationships), the backbone of *N. meningitidis* pConj is highly diverse compared with the gonococcal plasmid, indicating that pConj has a much longer evolutionary history with the meningococcus than the gonococcus. The identification of virtually identical plasmids in different clonal complexes (cc) of *N. meningitidis* indicates that the plasmid continues to disseminate within the meningococcal population. Evolutionary analyses reveal that *N. gonorrhoeae* has acquired pConj from *N. meningitidis* on at least two occasions. The first acquisition was followed by clonal expansion of lineages containing the plasmid and its spread to other gonococcal lineages. In contrast, the second introduction of pConj into the gonococcus was associated with a pConj variant with an altered component of its T4SS, TrbL, which reduces the conjugation efficiency, potentially explaining why this pConj variant is largely restricted to a single gonococcal lineage. In summary, pConj, an important driver of plasmid-mediated AMR, evolved in *N. meningitidis*, acquired resistance elements from streptococci, and spread into and proliferated in *N. gonorrhoeae*. This mirrors the acquisition by the gonococcus of p*bla* from another pathogenic species, *Haemophilus ducreyi* (27). Our findings also illustrate that, while chromosomal resistance arises through integration of sequences from commensal *Neisseria* (2, 3), plasmids in *N. gonorrhoeae* originally arose by transfer from pathogens. Due to the threat that AMR poses to treatment of gonococcal disease, AMR surveillance of the meningococcus, as well as commensals, is necessary to predict the spread and impact of AMR in pathogenic *Neisseria*.

## RESULTS

### Sporadic acquisition of p*bla* and pCryp by *N. meningitidis* from the gonococcus

To determine the prevalence of the gonococcal plasmids, pCryp, pConj, and p*bla*, in the meningococcus, we examined WGS of available *N. meningitidis* isolates on PubMLST (*n* = 19,951, Table S1). Only two meningococcal isolates in the database carry p*bla*, with both having the p*bla*.1/African variant of this plasmid. Isolate 54730 from clonal complex (cc) ST-5 was recovered from a patient with IMD in Chad during 2013, while isolate 54823 (cc ST-11) was isolated from a patient from Burkina Faso in 2012. Both isolates also carry pCryp but not pConj. The differences in the ccs, countries, and years, and the lack of pConj that could mobilize p*bla* suggest that the p*bla* was likely to have been acquired from the gonococcus through independent HGT events.

pCryp is the most common plasmid in the gonococcus, present in over 90% of isolates (6, 28). We examined meningococcal WGS for pCryp and found only 23 of 19,951 *N. meningitidis* isolates containing this plasmid. pCryp is recovered from several ccs, i.e., ST-11 (*n* = 8), ST-5 (*n* = 5), ST-41/44 (*n* = 4), ST-269 (*n* = 2), ST-23 (*n* = 1), and ST-10217 (*n* = 1) and from different locations, i.e., Chad (*n* = 5), Canada (*n* = 4), UK (*n* = 4), USA (*n* = 4), Sweden (*n* = 2), and Burkina Faso, Netherlands, Nigeria, and Spain (1 each).

**Fig 1:**
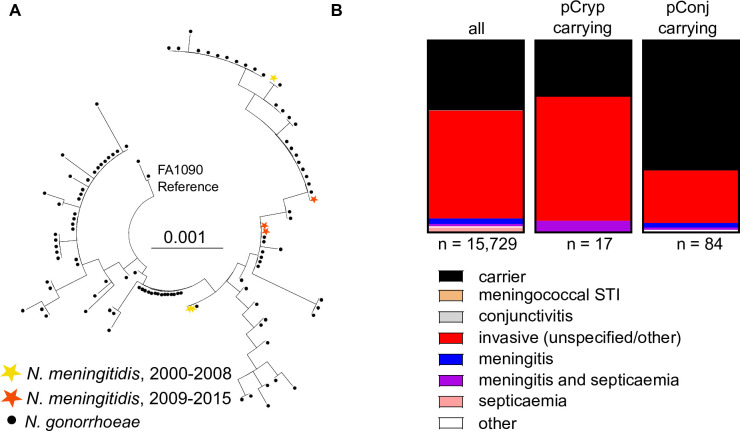
Evolutionary relationship between pCryp from pathogenic *Neisseria* and frequency of plasmids in isolates recovered from individuals with IMD or carriage. (**A**) Phylogenetic tree of pCryp from isolates of *N. meningitidis* and *N. gonorrhoeae*. Raw reads were downloaded from the ENA then assembled with SNIPPY. Trees were constructed with RAXML-NG and visualized with MEGA; isolates colored according to species (for *N. gonorrhoeae*) or year of isolation (for *N. meningitidis*). (**B**) Disease status data for all meningococcal strains in the database (where available) and for pCryp-carrying and pConj-carrying strains.

To determine relationships between pCryp in *N. gonorrhoeae* and *N. meningitidis*, we performed phylogenetic analysis of the plasmid in both species. Only isolates with raw reads available on the European Nucleotide Archive were included due to problems with *de novo* pCryp assembly on PubMLST (*n* = 8 *N*. *meningitidis* and *n* = 111 *N*. *gonorrhoeae* pCryp). The WGSs used for pCryp assembly were filtered based on length of low coverage sequences (<1000 bp), resulting in six *N. meningitidis* and 105 *N. gonorrhoeae* plasmids (Table S2). The sequence of pCryp from FA1090 was used as the reference. Results show that pCryp is highly conserved and plasmids in the gonococcus are indistinguishable from those from *N. meningitidis*, indicating that, similar to p*bla*, the meningococcus likely acquired the plasmid from the gonococcus. The lack of clustering of meningococcal pCryp in the tree and its occurrence in diverse strains indicate that pCryp has been transferred on multiple independent occasions (Fig. 1A). Furthermore, pCryp is found in carriage and IMD isolates in proportions similar to all meningococcal strains in the database (*P* = 0.6230, odds ratio 0.7263, Fig. 1B).

### pConj is more commonly found in *N. meningitidis* carriage than disease isolates

We found that pConj is the commonest gonococcal plasmid in *N. meningitidis*, with 104 of 19,951 (0.52%) meningococcal isolates carrying this plasmid. Although pConj is associated with pCryp in *N. gonorrhoeae* (26), none of the meningococcal isolates harboring pConj had pCryp. Meningococcal isolates carrying pConj were significantly more likely to be obtained from carriage rather than IMD compared with the other *N. meningitidis* isolates in the database (*P* < 0.0001, odds ratio 3.708, 95% CI: 2.338 to 5.848, Fig. 1B), suggesting that the plasmid imposes fitness costs during the development of disease or is beneficial during carriage.

Certain meningococci carry a functional Cas9 system which can reduce HGT by transformation (29). Therefore, we determined whether there is an association between pConj carriage and the presence of this CRISPR-Cas systems in *N. meningitidis* (*neis2566–2568* in PubMLST). Results show that 50% (52/104) of pConj carriage strains also carry the CRISPR-Cas locus, while 44.3% (8,794/19,847) of strains without pConj carry the CRISPR-Cas locus (*P* = 0.2764, Fisher’s exact test). Therefore, pConj carriage by the meningococcus is not associated with presence or absence of *neis2566–2568*.

### *pConj* has been acquired by the gonococcus from *N. meningitidis* at least twice

**Fig 2:**
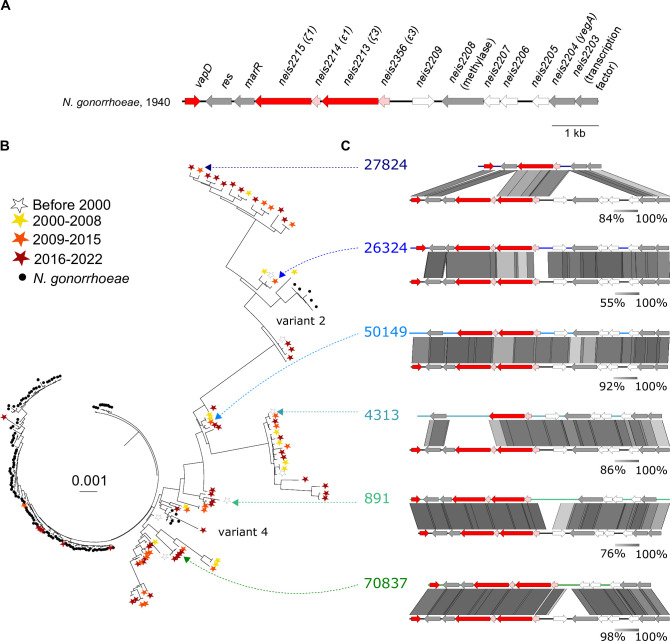
Variation in the GL region of meningococcal pConj. (**A**) The GL region of pConj from the earliest available isolate (78639 from 1940). The GL region refers to the genetic locus from *vapD* to *neis2203* (inclusive). (**B**) Representative phylogenetic tree of pConj from isolates of *N. meningitidis* and *N. gonorrhoeae*. Isolates are colored according to species (for *N. gonorrhoeae*) or years (for *N. meningitidis*) as shown. Meningococcal plasmids carrying *tetM* are indicated with a green arrow. (**C**) GL region from six representative meningococcal plasmids (27824, 26324, 50149, 4313, 891, and 70837, with their positions on the phylogenetic tree are indicated). GL regions are shown using EasyFig. ORFs colored: red, toxin; pink, antitoxin; gray, gene with known function; white, gene of unknown function.

A maximum likelihood tree was constructed of meningococcal pConj from strains with a known year of isolation and with the plasmid sequence on a single contig (*n* = 89 out of 104 plasmids), together with a selection of gonococcal pConj (*n* = 126) including examples of all seven pConj variants (26) (Table S3); the *tetM*^+^ Tn*916* transposon was excluded as HGT can distort evolutionary relationships (30). The resulting tree was run through ClonalFrameML to further adjust for recombination which is frequent in *Neisseria* spp (31). This revealed that meningococcal pConj exhibited far greater variation than the plasmid in *N. gonorrhoeae* particularly in the GL region (Fig. 2A).

Analysis of pConj-carrying isolates demonstrates that pConj is found in multiple clonal complexes (Table S4). Furthermore, closely related pConj are carried by meningococcal isolates belonging to different clonal complexes and isolated in different years. For example, pConj similar to that from isolate 27824 is also carried by isolates belonging to ccs ST-32, ST-35, ST-41/44, ST-178, ST-1157, and ST-4821; this was also seen with pConj from isolate 4313 (Table S4). The occasional presence of pConj in a range of clonal complexes indicates that the plasmid is sustained in *N. meningitidis* by low-level transmission. This differs from gonococcal carriage of pConj — carriage of pConj in *N. gonorrhoeae* is highly prevalent in certain lineages, suggesting that there is transmission of the plasmid within gonococcal populations (6).

Inspection of *N. meningitidis* pConj sequences revealed that the regions encoding the T4SS and factors involved in replication and partitioning do not exhibit large-scale changes. This does not include the GL region, which consists of genes from *vapD* to *neis2203*, inclusive (Fig. 2A). Occasionally, plasmids exhibit ambiguous sequencing in these regions (such as in isolates 27586, 92474, and 115472); however, these changes do not affect the overall phylogenetic tree for *N. meningitidis* pConj (Fig. 2B). Instead, variation in *N. meningitidis* pConj mostly occurs as deletions and insertions in the GL region. For example, three versions of meningococcal pConj in our data set carry distinct *tetM* insertions in the GL region (isolates 86009, 96516, and 115654) (Fig. 2B; Fig. S1), indicating that the plasmid in *N. meningitidis* has acquired this resistance element on multiple occasions. The GL region also differs through the presence of *neis2209*, a gene of unknown function (e.g., isolate 70837), as well as replacement of the orphan toxin *vapD* with sequence of unknown function (e.g., isolate 4313). Furthermore, some meningococcal pConj exhibit large deletions in the GL region compared to gonococcal pConj, including loss of *neis2206-2209*, encoding the *ε:ζ1* toxin-antitoxin system and a putative *marR* transcriptional regulator (e.g., isolate 27824) (9) (Fig. 2C). Additionally, we identified a meningococcal pConj that carries a gene of unknown function (*neis2211*) also found in *Kingella kingae* (Fig. S1).

**Fig 3:**
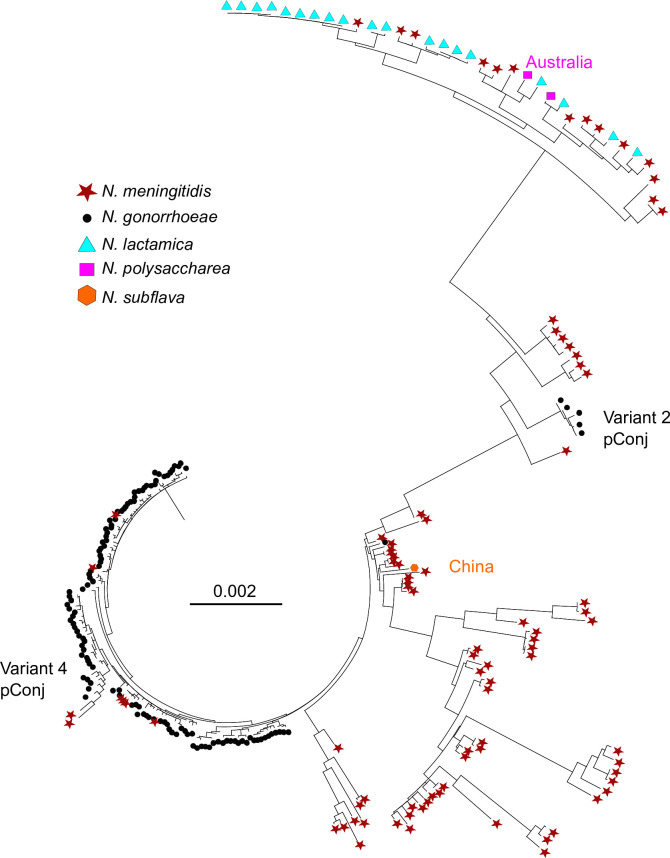
pConj has been acquired at least twice by *N. gonorrhoeae* from *N. meningitidis*. Construction of phylogenetic tree of pConj without the GL region, with the addition of pConj sequences from commensal *Neisseria* spp. Each species is shown using different shapes/colors as indicated. All commensal *Neisseria* spp. sequences were isolated from UK with the exception of two isolates as indicated. Gonococcal variant 2 pConj (indicated) is closely related to plasmids found in *N. meningitidis*. The position of variant 4 pConj is shown.

As deletions and recombination, such as those in the GL region, can distort phylogenetic analyses, we reconstructed pConj evolutionary trees excluding this region, i.e., with the plasmid backbone alone. Even without the GL region, meningococcal pConj is significantly more variable than gonococcal pConj (Fig. S2), indicating a long evolutionary relationship of the plasmid with *N. meningitidis* compared with the gonococcus. There are examples of pConj in diverse *N. meningitidis* isolates (i.e., isolates 60759, 97402, 79043, 55465, 55347, 80250, and 70850) in which the plasmid is indistinguishable from the highly conserved plasmids found in the gonococcus (Fig. 3; Fig. S2). Therefore, these plasmids likely share a recent common ancestor and all are probable descendants of pConj which was transferred from the meningococcus to *N. gonorrhoeae* and gave rise to most variants of the plasmid seen in the gonococcus.

Once the GL is excluded, variant 4 pConj is more closely related to gonococcal rather than meningococcal pConj, consistent with this variant being a descendant of other plasmids in *N. gonorrhoeae* with a portion of its GL region from meningococcal pConj. In contrast, even without the GL region, variant 2 pConj clusters with meningococcal versions of the plasmid rather than other variants of the plasmid in the gonococcus. This indicates that variant 2 pConj, which is largely restricted to a single lineage (6), was derived from a second separate introduction of pConj into the gonococcus from *N. meningitidis*.

### pConj in commensal *Neisseria* spp. are related to meningococcal pConj

Given the genetic diversity of meningococcal pConj, we next determined whether pConj in non-pathogenic *Neisseria* are more closely related to meningococcal or gonococcal pConj. The plasmid was identified in isolates of *Neisseria lactamica* (*n* = 20/805), *Neisseria polysaccharea* (*n* = 3/67), and *Neisseria subflava* (*n* = 1/57) (Table S5). A phylogenetic tree was constructed including pConj from these commensal species containing pConj on a single contig (*n* = 22/24); the GL region was excluded from analysis as above. Results show that pConj from commensal *Neisseria* spp. are most similar to meningococcal pConj (Fig. 3), in particular, those with large deletions in the GL region with loss of genes *neis2206-2209*, the *ε:ζ1* toxin–antitoxin system, and the putative *marR* transcriptional regulator (Fig. 3). However, it is difficult to draw conclusions from these results as, with the exception of two isolates (from China and Australia, Fig. 3), all plasmids from commensal species were obtained from individuals in the UK; thus, results could be distorted by opportunistic sampling.

### The *trbL* allele in variant 2 pConj is associated with reduced conjugation

**Fig 4:**
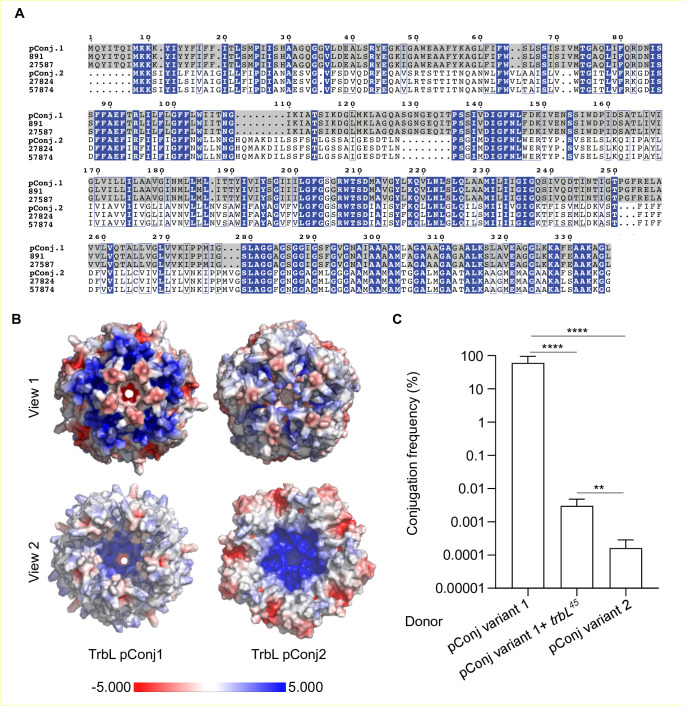
Different TrbL alleles affect the frequency of conjugation. (**A**) Alignment of TrbL from different meningococcal plasmids (isolates indicated) and from variants 1 and 2 of pConj (denoted as pConj.1 and pConj.2, respectively). Alignments were first carried out with COBALT and then visualized with ESprit. Identical residues are shown in white on blue background, residues with a similarity score higher than 0.7 are framed and colored blue; all remaining residues are shown in black. Residues conserved between pConj variant 1 sequences are shown on a gray background. (**B**) AlphaFold prediction of the structure of pentameric TrbL from variant 1 and 2 pConj using pConj.1 and pConj.2 sequences in (**A**). The structure is colored using the Adaptive Poisson-Boltzman Solver Electrostatics Plugin, where negatively and positively charged regions are shown in red and blue, respectively. (**C**) Conjugation frequency of donors with variant 1 or 2 pConj, or with an isogenic variant 1 pConj carrying the *trbL* allele found in variant two plasmids.

The presence of allele 45 of *trbL* (*trbL^45^*) is a defining feature of variant 2 pConj (6). VirB6 in *Agrobacterium tumefaciens*, a TrbL homolog, is an inner membrane component of the T4SS (32, 33) that is involved in transfer of the DNA:relaxase complex during conjugation (32, 34). We aligned the amino acid sequences of different TrbL alleles from pConj. Results indicate that *N. meningitidis* TrbL falls into at least two groups: (i) TrbL highly related to those in variant 1 gonococcal pConj and (ii) those similar to TrbL from variant 2 pConj (Fig. 4A). To establish whether these differences have functional consequences, we predicted structures of the TrbL from gonococcal pConj variants 1 and 2 by AlphaFold. The homolog of TrbL in *A. tumefaciens*, VirB6, is thought to form a channel through which DNA translocates during conjugation (35). In the conjugative R388 plasmid from *Escherichia coli*, the TrbL homolog, TrwI, forms a pentameric complex (36) Therefore, we performed AlphaFold predictions assembling TrbL into a pentamer. AlphaFold predicted that TrbL from variant 1 pConj forms a complex with a relatively open channel, while the TrbL complex predicted from variant 2 pConj forms a relatively closed channel which might impair the passage of DNA during conjugation (Fig. 4B; see Fig. S3 for confidence of prediction).

We therefore determined whether the different *trbL* alleles affect conjugation frequency. We performed isogenic matings between *N. gonorrhoeae* FA1090∆*pilD:ermC* donors containing variant 1 or 2 pConj, or variant 1 pConj with *trbL* from variant 2 introduced into its native site; the recipient in all experiments was FA1090-WT∆*pilD:aph (3*). All donors and recipients were *pilD* mutants to prevent plasmid transfer by transformation. Results demonstrate that variant 2 pConj has fours order of magnitude lower conjugation frequency compared to variant 1 pConj (*P* < 0.0001); additionally, introduction of *trbL45* from variant 2 pConj into a variant 1 plasmid significantly lowered the conjugation frequency (*P* < 0.0001 *vs*. wild-type variant 1 pConj, Fig. 4C). This is consistent with differences in *trbL* contributing to differences in conjugation rates observed for these pConj variants.

## DISCUSSION

The evolution and spread of resistance plasmids in pathogens have made treating bacterial infection increasingly difficult and contributed significantly to the AMR crisis (37, 38). While bacteria can acquire plasmids from distant or related species by conjugation (39, 40), the origin of plasmids can be difficult to trace. Plasmids often undergo recombination, making it difficult to identify ancestors and predict evolutionary pathways using current informatics tools (41). *N. gonorrhoeae* pConj is a highly conserved plasmid that first arose in the preantibiotic era (26, 42); the earliest isolate in PubMLST with pConj dates from 1940, but there are only a limited number of WGS from isolates predating this. Its spread ended the use of tetracyclines for gonococcal disease (43) and promoted resistance against β-lactams mediated by p*bla*. Furthermore, pConj is likely to undermine efforts to control STIs through doxycycline post-exposure prophylaxis (doxy-PEP) (7) as *tetM* confers high-level doxycycline resistance (6, 44). This could have serious consequences as dissemination of pConj in the meningococcus and gonococcus would promote the spread of p*bla* (11), potentially undermining the use of β-lactams to treat infections caused by these important human pathogens.

Here we show that pConj in *N. meningitidis* is highly diverse, consistent with a long evolutionary history with this pathogen. This contrasts with the conservation of pConj in *N. gonorrhoeae*, demonstrating that it was acquired more recently by the gonococcus from the meningococcus. Spread of the plasmid in the gonococcus, which occurs at high frequency by conjugation (45), and clonal expansion of lineages carrying the plasmid (by conferring a selective advantage in the presence of tetracycline/doxycycline, +/- in the genitourinary tract) have led to it become highly prevalent in the gonococcus, with around a third of all isolates carrying pConj. Our data indicate that variant 2 pConj was more recently acquired from the meningococcus. Gonococcal isolates with this variant were only identified from 2010 onwards (Table S3) but may have been present in gonococci before this time and not found due to limited availability of WGSs prior to this date. Of note, variant 2 pConj is mostly found in a single lineage, lineage 364 (previously lineage 151) (6). Although this might reflect its recent entry into *N. gonorrhoeae*, we demonstrate that the conjugation frequency of this variant is lowered by almost four orders of magnitude compared with variant 1 pConj, and this is consistent with different *trbL* alleles affecting the rate of transfer. Although pentameric TrbL from variant 2 plasmids is predicted to form a closed structure (Fig. 4), this might not represent its conformation when it is interacting with other partners in an active T4SS.

Closely related variants of pConj are found in a range of *N. meningitidis* clonal complexes from diverse geographic locations, indicating that the plasmid is sustained by transmission within this species, with little evidence of clonal expansion of isolates containing the plasmid (Tables S3 and S4). This suggests that the plasmid could be less stable in the meningococcus than in the gonococcus, which did not lose pConj after more than 160 generations (26). Additionally, pConj is associated with carriage of the meningococcus rather than IMD (Fig. 1B), consistent with previous case reports (21, 46). This contrasts with pCryp, which is evenly distributed among colonizing and disease-causing isolates (Fig. 1A). Therefore, pConj might impose fitness costs on bacteria especially during IMD but also during colonization, potentially explaining its relatively low prevalence in the meningococcus compared to *N. gonorrhoeae*. Alternatively, defence systems might limit the acquisition of pConj by strains that cause IMD. However, pConj was found in strains belonging to hypervirulent lineages (e.g., ST41/44) and there was no association between pConj carriage and the meningococcal CRISPR-Cas locus that limits transformation (29). Further work on characterizing systems that might limit HGT could offer insights into the distribution of plasmids in *Neisseria* spp.

Meningococcal pConj exhibits large-scale changes within its GL region (Fig. 2). It is possible that the GL region affects interactions with chromosomally derived factors and influences host range. For example, toxins encoded by TA systems on the GL region could target factors in particular species or lineages, while the MarR family transcription factor (*neis2216*) could regulate pathways in *N. gonorrhoeae* but not in *N. meningitidis*. Further experiments are underway to define the contribution of the GL region on bacterial fitness and survival in different host niches.

Initial analysis suggested that variant 4 of gonococcal pConj clusters with meningococcal plasmids; however, subsequent removal of the GL region from analysis demonstrates that variant 4 plasmids are closely related to other gonococcal pConj variants. This suggests that variant 4 pConj is a hybrid of gonococcal and meningococcal plasmids. It also validates our use of pConj backbone genes to determine the evolutionary relationships between the plasmid from different *Neisseria* spp. as the variation in GL, through gene gain/loss, is a confounding factor when building phylogenetic trees.

pConj is also carried by non-pathogenic *Neisseria* spp., such as *N. lactamica* and *N. polysaccharea*, which are closely related to the pathogenic species (47). Of note, pConj in the commensals is indistinguishable from plasmid variants circulating in the meningococcus, suggesting exchange of pConj between *N. meningitidis* and commensal *Neisseria* spp. which inhabit the human oropharyngeal tract. Interestingly, apart from one example of pConj in *N. subflava* isolated in China, the plasmids in commensals cluster with a single group of meningococcal pConj (Fig. 3). However, it is difficult to interpret the significance as we identified a limited number of commensals with pConj, most of which were submitted to PubMLST from a single location (Southampton, UK). Therefore, more WGSs of commensal isolates are needed to appreciate the population biology of pConj in commensal *Neisseria* spp.

The evolution of pConj in *N. meningitidis* has important implications. It is widely appreciated that chromosomally mediated AMR in the gonococcus arose through HGT between this pathogen and commensals in the upper respiratory tract (1–3, 48). Our findings highlight how a mobile genetic element that is central for plasmid-mediated AMR in the gonococcus was also acquired from a resident in the upper airway. However, in the case of pConj, the plasmid was transferred from pathogen to pathogenon in at least two occasions. The plasmid itself has acquired *tetM* from streptococci, an abundant component of the upper respiratory tract, on at least multiple occasions, evidenced by the different *tetM* alleles and different insertion sites in the GL region (49). Therefore, surveillance of bacteria inhabiting the upper respiratory tract is required as they act as reservoirs for vehicles of AMR as well as the source of AMR genes for the WHO urgent threat list pathogen, *N. gonorrhoeae* (1).

**Fig 5:**
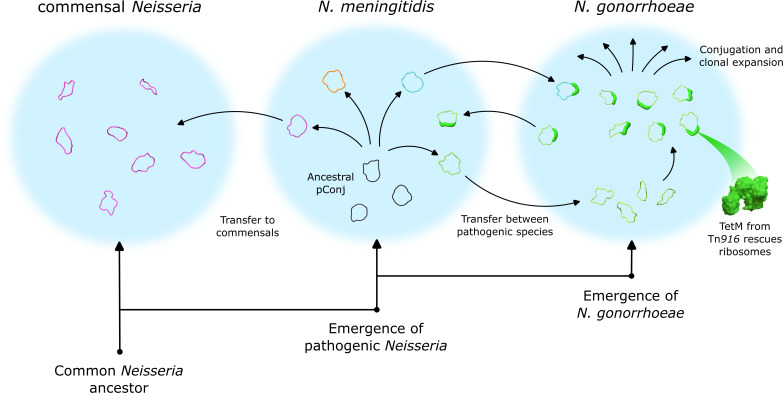
pConj evolved in *N. meningitidis* and spread to *N. gonorrhoeae* and commensal *Neisseria* spp. Ancestral pConj likely first emerged in *N. meningitidis* where it has evolved and become genetically diverse. The plasmid has been transferred from the meningococcus into *N. gonorrhoeae* on at least two occasions and acquired a partial copy of Tn*916* with *tetM,* conferring resistance against tetracycline and doxycycline. In the gonococcus, pConj spread by conjugation into different lineages which have undergone clonal expansion, probably driven by exposure to antibiotics. pConj has also likely spread from the meningococcus to commensal *Neisseria* spp., resulting in another reservoir of plasmids. Increasing tetracycline useis likely to increase pConj prevalence in all *Neisseria* spp.

Although currently pConj is relatively uncommon in *N. meningitidis*, the widespread use of doxycycline, through the implementation of doxy-PEP (7), is likely to promote the spread of *tetM*^+^pConj in *Neisseria* spp. Given the ability of pConj to promote p*bla* spread and the association between these two plasmids in the gonococcus (6, 11, 16), doxy-PEP could increase the prevalence of p*bla* as well as pConj in both *N. gonorrhoeae* and *N. meningitidis*, with the latter jeopardizing treatment of IMD with β-lactams (46). The combination of pConj continually evolving in *N. meningitidis*, commensal *Neisseria* spp. acting as reservoirs for AMR plasmids*,* doxycycline pressure from *N. gonorrhoeae* treatment, and doxy-PEP and HGT between all *Neisseria* spp. (Fig. 5), suggests that widespread plasmid-mediated resistance in both pathogenic and commensal *Neisseria* spp. could emerge as a major threat to public health in the future.

## MATERIALS AND METHODS

### Analysis of plasmid carriage

Analysis of plasmids in *N. meningitidis* was performed using publicly available WGS on the PubMLST database (https://pubmlst.org/) (25), comprising 19,951 *N*. *meningitidis* isolates (Table S1, accessed 4 May 2023). Isolates (*n* = 929) belonging to commensal *Neisseria* spp. with evidence of pConj carriage are shown in Table S5, accessed 18 June 2023. The presence of *neis2960* was used to identify p*bla*, while *traM (neis2241*) and *trbC (neis*2230) were used to detect the presence of pConj; *repA (neis2952*) and *mobC* (*neis2951*) were used to indicate the presence of pCryp [(6, 8, 26) and Table S6].

### Phylogenetic analyses

For construction of pCryp phylogenetic trees, raw reads were downloaded from the European Nucleotide Archive database (https://www.ebi.ac.uk/ena/browser/home) due to incomplete assembly of pCryp on single contigs in PubMLST. Raw reads were included from 126 gonococci carrying pConj used previously (26) and all *N. meningitidis* isolates carrying the plasmid (Table S2). Single nucleotide polymorphisms were identified with SNIPPY (version 4.6.0) using the FA1090 pCryp sequence as reference. Core alignment files generated by SNIPPY using the default parameters were first filtered for low coverage, where sequences carrying >1,000 bp of low coverage were excluded. The final set of sequences was then inputted into RAXML-NG (50) for construction of phylogenetic trees with an initial 20 maximum likelihood starting trees, followed by generation of 1,000 trees or until convergence was reached, whichever came first. For each alignment, phylogenetic trees were generated three times independently (i.e., with three individual seeds). The final trees were visualized with MEGA (version 11.0.11) and a representative tree was selected. To compare between individual plasmids, we inputted sequences into EasyFig version 2.2.5. All alignments were performed with tblastx, with a threshold of 100 bp.

pConj sequences were downloaded from PubMLST, only including sequences from isolates with a known year of isolation and assembled in a single contig to ensure high-quality data. All isolates used for phylogenetic analyses of pConj are listed in Table S3. Gonococcal pConj sequences were used for the construction of trees as previously described (26). Tn*916* with *tetM* is found in some pConj variants (51) and was removed from evolutionary analyses which can be confounded by HGT. Separately, we re-constructed evolutionary pathways after eliminating the GL region (*neis2203* to *vapD*) by first aligning *trbN* and *traC* (which flank the GL region), then removing intervening sequences. Sequences were aligned with the Multiple Alignment using Fast Fourier Transform (MAFFT) program (52) on Galaxy (usegalaxy.org) then analyzed with RAXML-NG version 1.1.0 using the GTRGAMMA model of nucleotide substitution for phylogenetic tree construction (50). All alignments were then inputted into ClonalFrameML (version 1.12) to generate phylogenetic trees which account for small-scale recombination (31).

### Structure predictions

Predictions of the structure of pentameric TrbL were performed with AlphaFold version 2.3.1 (53) and visualized with PyMol (54); truncated TrbL (i.e., amino acids 1–336 for TrbL from variant 1 pConj and amino acids 1–330 for TrbL from variant 2 pConj) lacking transmembrane domains (Table S7) were used for AlphaFold predictions. Structures were colored based on the Adaptive Poisson-Boltzmann Solver Electrostatics Plugin (APBS electrostatics plugin visualization tool), which shows the charge distribution, as well as B-factor, which shows the confidence of predictions.

### Construction of mutants

All primers used in this study are listed in Table S8. To introduce different *trbL* alleles into their native site on pConj, we amplified sequences upstream and downstream of *trbL* with primers 506/507 and 510/511, respectively, using variant 1 pConj as the template. *trbL* allele 45 was amplified from variant 2 pConj with primers 508/509. These sequences were fused with the *galK-aph (3*) cassette amplified with primers 92/KanF (45). For the subsequent markerless deletion, sequences upstream and downstream of the *galK-aph (3*) cassette were amplified with primers 506/512 and 511/513, respectively, before the final construct was amplified by fusing the two fragments with overlap PCR using primers 506/511. Products were used to transform *N. gonorrhoeae* as described previously (45).

### Bacterial strains and conjugation

FA1090 donor (∆*pilD::ermC* and *tetM*^+^pConj) and recipient strains (∆*pilD::aph (3*), Table S9) were grown overnight on solid GCB media (45) at 37°C in the presence of 5% CO_2_, then used to inoculate 5 mL of liquid gonococcal base media (GCBL) +1% Vitox (Oxoid) at an initial OD_600_ of 0.1. Cultures were incubated until the OD_600_ reached 0.5–0.8. Donors and recipients were then diluted to 10^7^ CFU/mL and mixed at a 1:1 ratio and to 10 µL of the mixture spotted on GCB agar plates and left to dry. Plates were incubated for 24 hr at 37°C, 5% CO_2_, before bacteria were harvested and resuspended in 1 mL GCBL. Bacteria were serially diluted and spotted onto plain GCB plates (to measure the total bacterial population) or on plates with erythromycin (1 µg/mL) and tetracycline (2 µg/mL) to select for the donor or with kanamycin (50 µg/mL) to select for the recipient or plates with both kanamycin and tetracycline to select for transconjugants. Conjugation frequencies were calculated as the percentage of recipients that were transconjugants (see Table S10 for raw data).

### Statistical analyses

Truncated TrbL sequences from pConj variant 1 (id: 36291), pConj variant 2 (id: 36284), and meningococcal pConj (id: 891, 27587, 27824, 57874) were aligned using COBALT (55) with RPS BLAST to guide alignment and the following parameters: Gap Penalties: Opening = −11, Extension = −1, End-Gap Penalties: Opening = −5, Extension = −1, Constraint E-value: 0.005, Word Size: 4, Max Cluster distance: 0.8, Alphabet: SE-B15. The alignment was visualized with ESprit 3.0 (56). Statistical significance was assessed using either Fisher’s exact test, odds ratio (Baptista-Pike), or one-way ANOVA with the appropriate multiple comparisons. Log transformation and tests for the normality of data were carried out as required. All statistical analyses were performed with GraphPad PRISM v 9.0.

## Data Availability

All isolates used for analysis and construction of phylogenetic trees are as listed in the respective supplemental tables. Sequencing data for these isolates can be found on PubMLST. The pCryp sequence of the lab’s FA1090 which was used as the reference sequence is uploaded and is also available on PubMLST (id: 106187). Strains are available on request.

## SUPPLEMENTAL MATERIAL

Supplemental information msphere.00441-23-s0001.docx Fig. S1-3; Tables S7 to S9; captions for Tables S1 to S6 and S10.

Table S1 msphere.00441-23-s0002.xlsx *N. meningitidis* data set.

Table S2 msphere.00441-23-s0003.xlsx pCryp analysis data set.

Table S3 msphere.00441-23-s0004.xlsx pConj analysis data set.

Table S4 msphere.00441-23-s0005.xlsx Meningococcal isolates carrying pConj.

Table S5 msphere.00441-23-s0006.xlsx Data set of commensal *Neisseria* species with evidence of pConj carriage.

Table S6 msphere.00441-23-s0007.xlsx Percentage identity index of all alleles of *traM*, *trbC*, *repA*, and *mobC*.

Table S10 msphere.00441-23-s0008.xlsx Raw data and calculations of conjugation frequencies for all conjugation reactions.

ASM does not own the copyrights to Supplemental Material that may be linked to, or accessed through, an article. The authors have granted ASM a non-exclusive, world-wide license to publish the Supplemental Material files. Please contact the corresponding author directly for reuse.
