# Supplementary material for: Evolution and exchange of plasmids in pathogenic Neisseria
Source: mSphere. 2023 Oct 18;8(6):e00441-23. doi: 10.1128/msphere.00441-23 (PMC10732060; doi:10.1128/msphere.00441-23)
Supplement: Supplemental information — Fig. S1-3; Tables S7 to S9; captions for Tables S1 to S6 and S10. [file msphere.00441-23-s0001.docx]

**SUPPLEMENTARY INFORMATION**

**
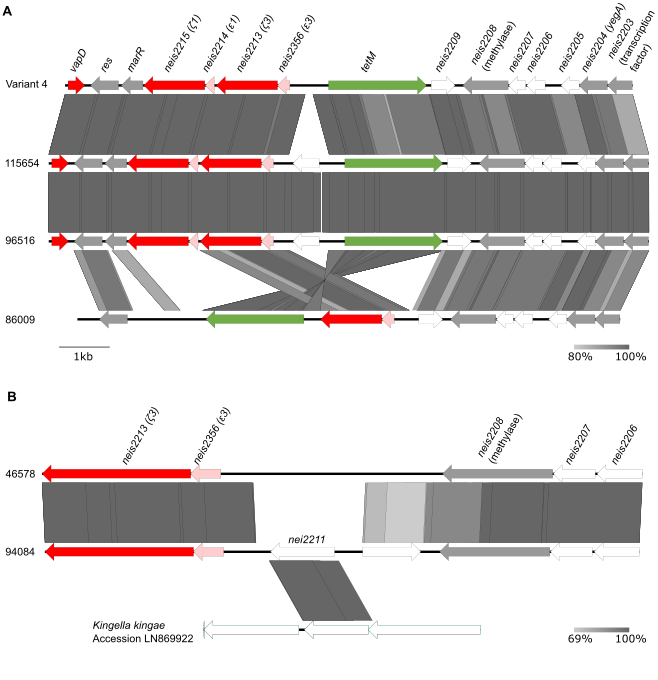
**

**FIG S1** Presence of *tetM* and *neis2211* in *N. meningitidis*. (A) Three meningococcal pConj carry *tetM*. The isolate and position of *tetM* insertion are shown. *neis2211* in meningococcal pConj shares similarity with a sequence in *Kingella kingae*. (B) Meningococcal pConj sequences of isolates 46578 and 94084 were downloaded from PubMLST was aligned with *Kingella kingae* chromosomal sequence from NCBI database using EasyFig.

**
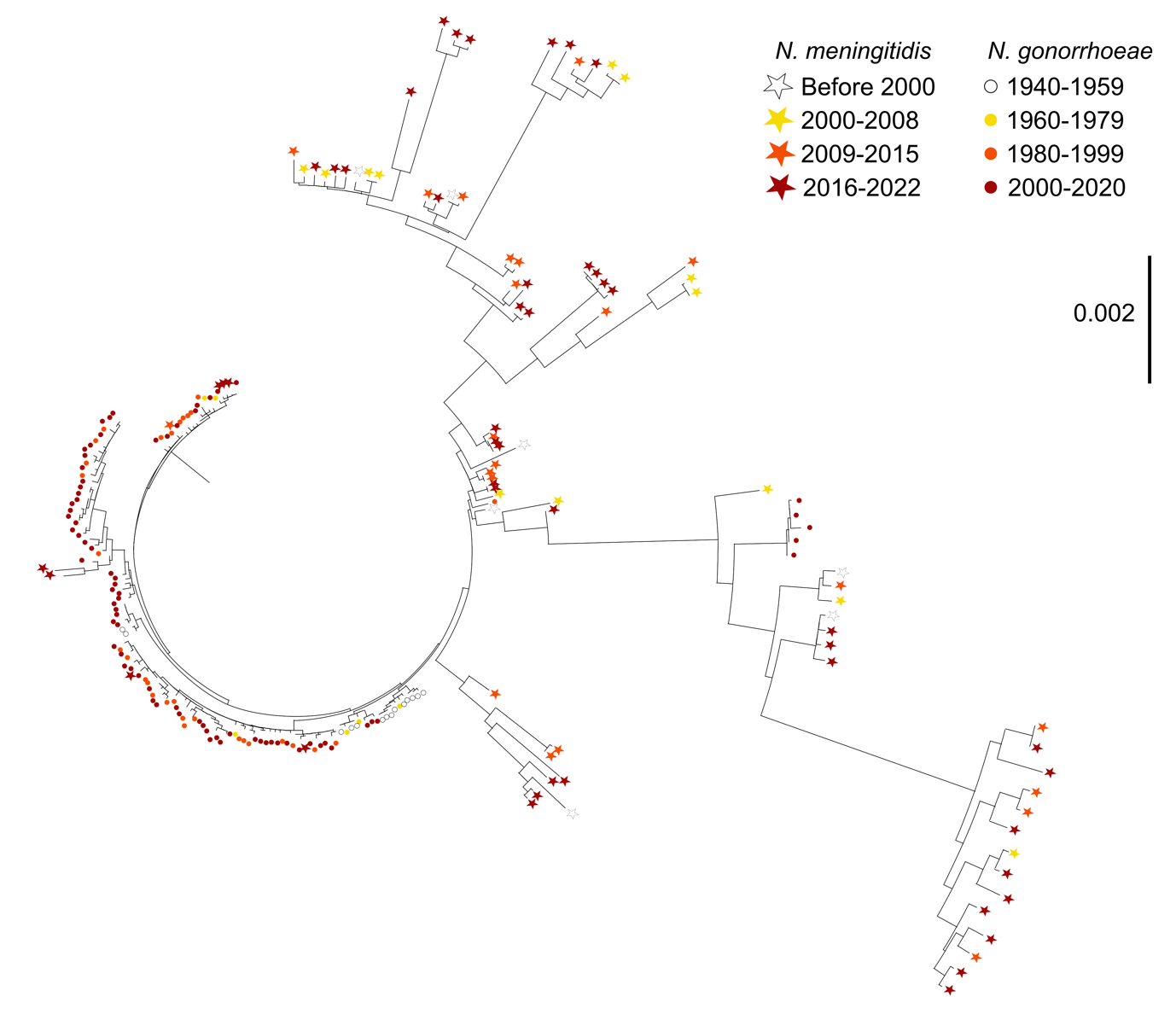
**

**FIG S2** Meningococcal pConj exhibits variation in its backbone. The GL region was removed from pConj, then sequences aligned with MAFFT before tree construction with RAXML-NG and ClonalFrameML. pConj from gonococci (circles) and meningococci (stars) are coloured according to their year of isolation.


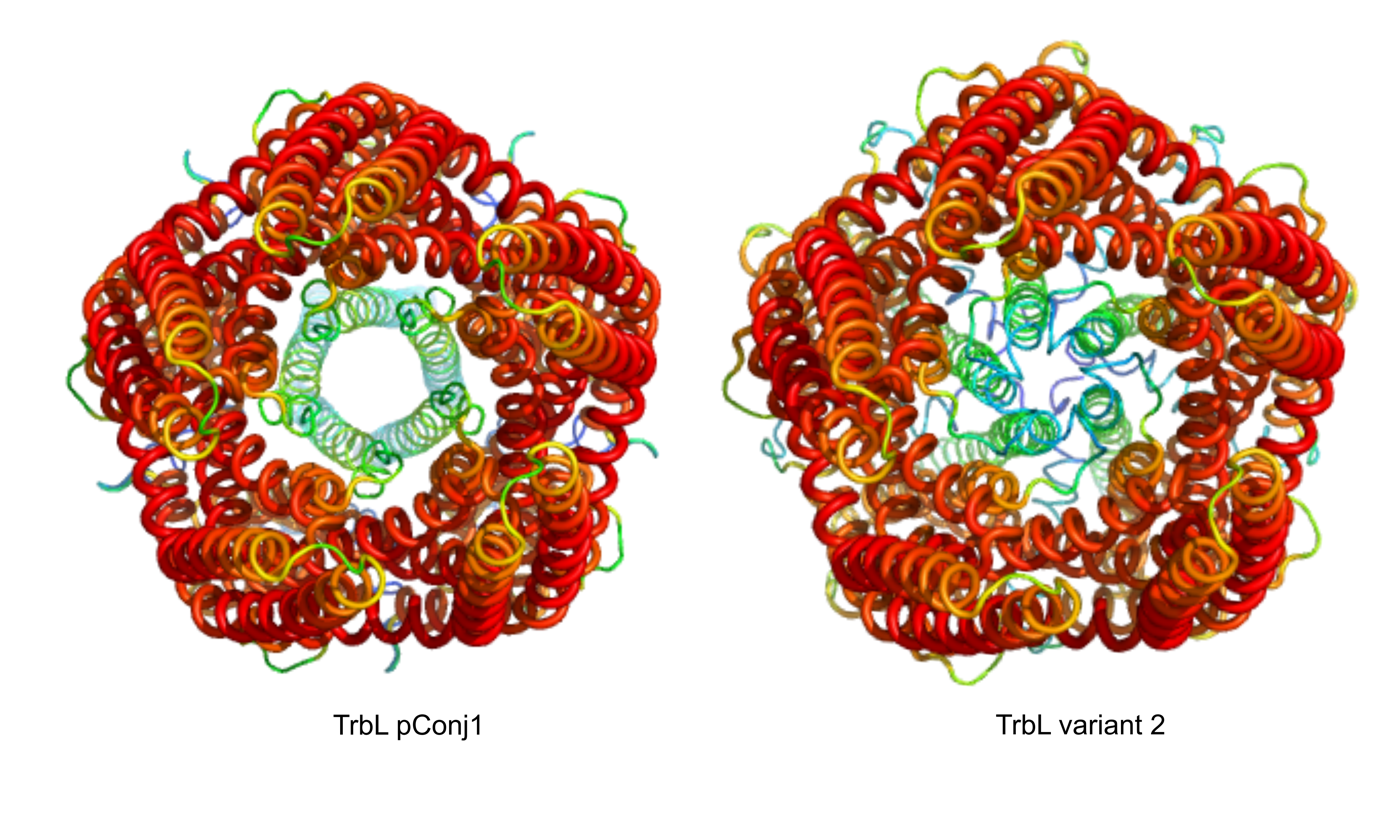


**FIG S3** Confidence of AlphaFold prediction of TrbL from variant 1 and 2 pConj. Predicted structures of pentameric TrbL are coloured according to B-factor; red shows highest confidence, while blue shows low confidence.

**Table S1.** *N. meningitidis* dataset

**Table S2.** pCryp analysis dataset

**Table S3.** pConj analysis dataset

**Table S4**. List of meningococcal isolates carrying pConj. Information on the clonal complex of each isolate is indicated, if known.

**Table S5.** Dataset of commensal *Neisseria* species with evidence of pConj carriage.

**Table S6.** Percentage identity index of all alleles of *traM*, *trbC*, *repA* and *mobC*.

| Source | Truncated amino acid sequence |
| --- | --- |
| TrbL from pConj variant 1 | MQYITQIMKKKYIYYFIFFITLSMPIISHAAGQGGVLDEALSRYEGKIGAWEAAFYKAGLFIFWSLSSISIVMTGAQLIFQRDNISSFFAEFTRLILFLGFFLWIITNGIKIATSIKDGLMKLAGQASGNGEQITPSSIVDIGFNLFDKIVENSSIWDPIDSATLIVIGLVILLILAAVGINMLLMLITTYIVIYSGIIILGFGSGRWTSDMAVGYLKQVLNLSLQLAAMILIIGIGQSIVQDTINTIGTPGFRELAVVLVQTALLVGLVVKIPPMIGSLAGGAGSGGIGSFGVGNAIAAAAMLAGAAAGAGAALKSLAVEAGGLKKAFEAAKAGL |
| TrbL from pConj variant 2 | MKKSIYILFIVAIGILLFIPDIANAESVGVFSDVQDRFEQAVSRTSTTITNQANWLFWVLAAISLVWTGITLVFRKGDISDFFAEFIRFIIFIGFFNWLLNNGHQMAKDILSSFSTLGSSAIGESDTLNPSGIMDIGFNLWERTYPSVSELSLKQIIPAYLIVIAVVIIVGLIAVNVLLLNVSAWIFAYAGVFVLGFGGSRWTSDIAISYFKQLLNLGLQILSMIIIVGIGKTFISVMLDKVSTFIFFDFVVILLCVIVLLYLVNKIPPMVGSLAGGFGNGGAGMLGGGAAMAAMAMTGGALMGAATALKAAGMEMAGAAKAFSAAKKGG |

**Table S7.** Amino acid sequences of truncated TrbL used

| **Primer** | **Sequence** |
| --- | --- |
| 506 | GCAAAACCAAGACCGTAGCC |
| 507 | GACCGTCGGGCATCTTTCCGCCGTCATTCCAAGAAAATAACTACTCTTTCCTTTTGTATG |
| 508 | TTCAATAGCTATAAATTATTTAATAAGTAAATGAAAAAGTCAATTTACATATTATTCATTGTAG |
| 509 | CCGACCAGCAATATGCAGATAAGCGTTTTTTTAAGCATTGTCGTCTCCTTCCG |
| 510 | AAAAACGCTTATCTGCATATTGCTG |
| 511 | TGGCGTTATTCCATTGCCTACC |
| 512 | AATGAATAATATGTAAATTGACTTTTTCATAAGAAAATAACTACTCTTTCCTTTTGTATGGC |
| 513 | ATGAAAAAGTCAATTTACATATTATTCATTGTAG |
| 92 | GGAATGACGGCGGAAAGATG |
| KanF | TTAGAAAAACTCATCGAGCATCAAATGAAACT |

**Table S8**. List of primers used

| *N. gonorrhoeae* strains | Reference |
| --- | --- |
| 44593 | (6) |
| FA1090-WT | Kind gift from Professor Ann Jerse |
| FA1090-WT∆*pilD::ermC* pConj^44593^ | This article |
| FA1090-WT∆*pilD::ermC* pConj^60755^ | This article |
| FA1090-WT∆*pilD::ermC* pConj^60755::^*^trbL^*^45^ | This article |
| FA1090-WT∆*pilD::kan* | (45) |

**Table S9**. List of strains constructed

**Table S10.** Raw data and calculations of conjugation frequencies for all conjugation reactions.
